# Association between homocysteine level and the risk of diabetic retinopathy: a systematic review and meta-analysis

**DOI:** 10.1186/s13098-018-0362-1

**Published:** 2018-08-02

**Authors:** Xunwen Lei, Guifeng Zeng, Yuemei Zhang, Qiang Li, Jinzhi Zhang, Zhenggang Bai, Kehu Yang

**Affiliations:** 1 The First Clinical Medical College of Lanzhou University, Lanzhou, 730000 China; 2 Evidence-Based Medicine Center, School of Basic Medical Sciences of Lanzhou University, Lanzhou, 730000 China; 3 Key Laboratory of Evidence-Based Medicine and Knowledge Translation of Gansu Province, Lanzhou, 730000 China; 4 Gansu Health Vocational College, Lanzhou, 730000 China; 5 School of Public Affairs, Nanjing University of Science and Technology, Nanjing, 210094 China

**Keywords:** Elevated homocysteine, Diabetic retinopathy, Systematic review, Meta-analysis

## Abstract

**Background:**

Previous studies have demonstrated that elevated homocysteine (Hcy) level represents an independent risk factor for macrovascular disease. However, the relationship between hyperhomocysteinemia and the progression of diabetic retinopathy in patients remains controversial. Hence, the purpose of this systematic review and meta-analysis was to explore any potential association between Hcy and the risk of diabetic retinopathy.

**Methods:**

PubMed, Embase, and the Cochrane Library databases were searched to screen studies that fulfilled the inclusion criteria from date of database inception to November 2017. The summary odds ratio (OR) with 95% confidence intervals (CIs) was used to calculate the pooled effect estimate for the relationship between Hcy and diabetic retinopathy risk. Sensitivity, subgroup analyses, and publication bias were also assessed.

**Results:**

Eleven studies involving a total of 2184 diabetic patients were included in the meta-analysis. The summary OR suggested that increased Hcy level in diabetic patients was associated with an increased risk of diabetic retinopathy (OR 1.62; 95% CI 1.29–2.03; p < 0.001). Although significant heterogeneity was detected among the included studies, the findings of sensitivity analysis remained statistically significant. Subgroup analyses found a significant association between Hcy and diabetic retinopathy in most subsets, but no significant association was found if the sample size was < 100, participants had type 1 diabetes mellitus, and the study quality was low.

**Conclusions:**

The findings of this study suggested that elevated Hcy level was associated with an increased risk of diabetic retinopathy, especially in type 2 diabetic patients. This finding may help diabetic patients to achieve effective management strategy to prevent the progression of diabetic retinopathy.

**Electronic supplementary material:**

The online version of this article (10.1186/s13098-018-0362-1) contains supplementary material, which is available to authorized users.

## Background

Diabetic retinopathy is a major vascular complication of diabetes mellitus (DM), often leading to low vision and blindness [1, 2]. It can become a major threat to public health in the future due to the increasing global prevalence of DM, which is projected to affect 438 million people by 2030 [3]. Diabetic retinopathy occurs in 70% of patients with diabetes for more than 15 years. A recent study indicated that the estimated prevalence of diabetic retinopathy was 28.5% among US adults [4]. The pathogenesis and etiology of diabetic retinopathy include poor glycemic control, vascular endothelial cell injury, hypercoagulability, ischemia and anoxia of retina, and genetic factors [5].

Homocysteine (Hcy) is a sulfur-containing amino acid formed by the demethylation of methionine. Plasma Hcy level is elevated in patients with diabetes, particularly those with type 2 diabetes as well as in pre-diabetic individuals with insulin resistance [6]. It is an emerging risk factor for diabetic retinopathy and cardiovascular disease that has become a research hotspot [7]. B vitamin is an essential vitamin for humans and an important cofactor of Hcy metabolism, which is commonly related to high circulating levels of Hcy. The effect of B vitamin supplementation on the progression of diabetic retinopathy in diabetic patients remains unknown, and requires a systematic review and meta-analysis.

A few studies have suggested that the level of plasma and vitreous Hcy is a risk factor for diabetic retinopathy [8–10], while others have refuted this link [11, 12]. Therefore, the present meta-analysis was performed to summarize currently available data to provide more robust evidence on the potential relationship between Hcy level and the risk of diabetic retinopathy.

## Methods

### Data sources, search strategy, and selection criteria

This meta-analysis was conducted according to the preferred reporting items for systematic reviews and meta-analysis guidelines [13]. All studies written in English that investigated the relationship between plasma Hcy level and diabetic retinopathy in diabetic patients were considered for this meta-analysis, regardless of the publication status (published, in press, or in progress). The PubMed, Embase, and Cochrane Library databases were independently searched by two authors to retrieve related original studies published before November 2017. The search terms used were as follows: “diabetic retinopathy,” “diabetic retinopathies,” “homocysteine,” and “Hcy”. Furthermore, the reference lists of all eligible studies were manually searched to identify any additional relevant studies.

The study selection was independently performed by two authors with a standardized approach, and any inconsistencies were resolved by group discussion and consensus. Studies that met the following inclusion criteria were included in this meta-analysis: (1) the study had an observational design; (2) the study reported the association of Hcy level with the risk of diabetic retinopathy; and (3) the study reported the sample size, diabetic retinopathy incidence or effect estimate. Studies that met any one of the following conditions were excluded: (1) article type: editorials, meta-analysis, and review articles; (2) did not report diabetic retinopathy (including proliferative and nonproliferative); and (3) duplication of published articles.

### Data collection and quality assessment

Data were independently extracted from each included study by two authors, and all disagreements were discussed and resolved with the help of the third author. The following information was recorded: first author’s name, publication year, country, sample size, mean age, percentage male, body mass index (BMI), diabetes mellitus types, cutoff value of Hcy, adjusted factors, and reported outcomes. The quality of selected studies was evaluated using the Newcastle–Ottawa Quality Assessment Scale (NOS) [14]. The maximal NOS score was 9 points, and studies ≥ 7 points were considered to be of relatively higher quality. The quality assessment was independently conducted by two authors, and information was examined and adjudicated by an additional author after referring to the original studies.

### Statistical analysis

Odds ratios and 95% confidence intervals (CIs) was used to assess the relationship between Hcy level and the risk of diabetic retinopathy in diabetic patients. The random-effects model was used to calculate summary ORs and 95% CIs for the high versus low Hcy levels [15, 16]. Heterogeneity among the included studies was quantified using the *I*-square (*I*^2^) value. *I*^2^> 50% was considered to represent significant heterogeneity [17]. We also performed a sensitivity analysis by removing each individual study from the meta-analysis [18]. Univariate meta-regression analysis based on sample size, mean age, percentage male, and BMI was used to explore the source of heterogeneity in estimates of the risk on diabetic retinopathy [19]. Subgroup analysis was conducted for diabetic retinopathy according to publication year, country, sample size, mean age, percentage male, BMI, diabetes mellitus types, and NOS score. Furthermore, ratio between subgroups was calculated to compare the differences between estimates of the two subsets, which were based on Student *t* distribution rather than normal distribution because of the small number of included studies [20]. Funnel plot, Egger and Begg tests were performed to evaluate the potential publication bias [21, 22]. All reported p values are two-sided, and p < 0.05 was regarded as statistically significant. All analyses were performed using STATA software package (version 10.0; Stata Corp, TX, USA).

## Results

**Fig. 1:**
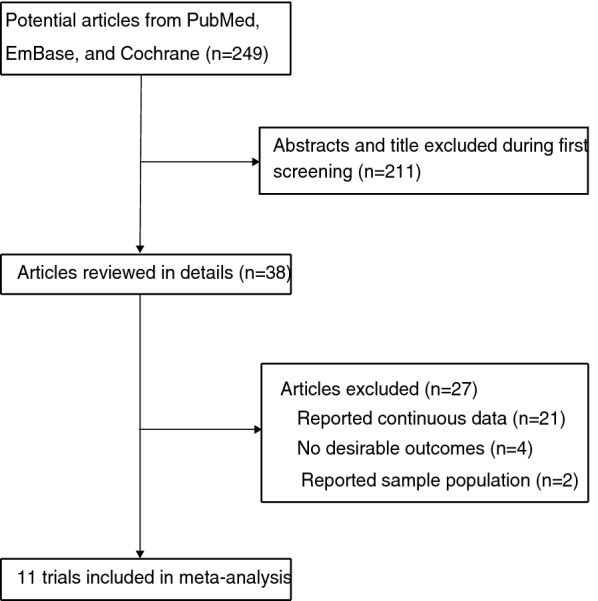
Flow diagram of the literature search and trial selection process

Initially 249 studies were extracted from PubMed, Embase, and the Cochrane Library databases, of which 211 studies were excluded after reviewing the titles and abstracts because they were unrelated to the topic or duplicates. By examining the full texts of the remaining 38 articles, 27 were excluded for including the same group of patients, reporting other outcomes, or without available data. A manual search of the reference lists of these studies did not yield any new eligible studies. The results of the study-selection process are shown in Fig. 1. Finally, 11 studies involving 2184 diabetic patients were included in this meta-analysis [3, 23–32].

The characteristics of the 11 included studies are shown in Table 1. The included studies were published between 1997 and 2014, and 25–625 diabetic patients were included in each study. Four of the included studies were conducted in Eastern countries [23, 28, 30, 31], and the remaining seven studies were conducted in Western countries [3, 23–27, 29, 32]. Furthermore, four studies included patients with type 1 diabetes mellitus [23, 24, 26, 32], five studies included patients with type 2 diabetes mellitus [3, 25, 29–31], and the remaining two studies included both type 1 and 2 diabetes mellitus [27, 28]. The mean age of patients in individual studies ranged from 43.8 to 69.4 years, the percentage male ranged from 41.9 to 78.6%, and mean BMI ranged from 24.2 to 29.6 kg/m^2^. NOS was used to evaluate the quality of each study, whereby two of the included studies had a score of 8 [24, 30], five studies had a score of 7 [3, 25, 26, 28, 32], and the remaining four studies had a score of 6 [23, 27, 29, 31].

**Table 1:** Baseline characteristics of studies included in the systematic review and meta-analysis

Study	Publication year	Country	Sample size	Mean age (years)	Percentage male (%)	BMI (kg/m^2^)	DM types	Cutoff of Hcy (μmol/L)	Adjusted factor	NOS score
Vaccaro [23]	1997	Japan	25	44.9	72.0	NA	1	10.0	Age, gender, blood pressure, creatinine, plasma cholesterol, smoking status	6
Hofmann [24]	1998	France	75	51.0	46.7	24.2	1	15.8	Age, gender, diabetes duration, diabetes control, blood pressure, BMI, plasma cholesterol	8
Hoogeveen [25]	2000	Netherlands	625	64.3	48.2	27.2	2	16.0	Age, gender, BMI, creatinine, plasma cholesterol	7
Buysschaert [26]	2000	Belgium	122	62.6	67.2	29.6	1	15.0	Age, gender, diabetes duration, hypoglycemia medication, blood pressure, triglyceride, LDL, HDL	7
Agullo-Ortuno [27]	2002	Spain	89	43.8	51.7	NA	Both	15.6 (men) and 13.9 (women)	Plasma cholesterol, triglyceride, LDL, HDL	6
Goldstein [28]	2004	Israel	335	69.4	51.3	NA	Both	15.0	Age, blood pressure, other complications	7
de Luis [29]	2005	Spain	155	64.6	41.9	29.6	2	15.0	Age, blood pressure, BMI, creatinine, plasma cholesterol	6
Golbahar [30]	2008	Bahrain	254	56.1	54.3	25.5	2	12.0 (men) and 15.0 (women)	Age, gender, diabetes duration, diabetes control, BMI, HDL, smoking status	8
Satyanarayana [3]	2011	US	300	55.1	56.1	24.4	2	12.0	Age, hypoglycemia medication, plasma cholesterol, low density lipoprotein	7
Sato [31]	2013	Japan	84	62.0	78.6	25.3	2	10.0	Metformin, eGFR, and B12	6
Bulum [32]	2014	Croatia	100	47.0	54.0	25.0	1	15.0	Age, eGFR, blood pressure, triglyceride	7

*LDL* low-density lipoprotein, *HDL* high-density lipoprotein, *BMI* body mass index

**Fig. 2:**
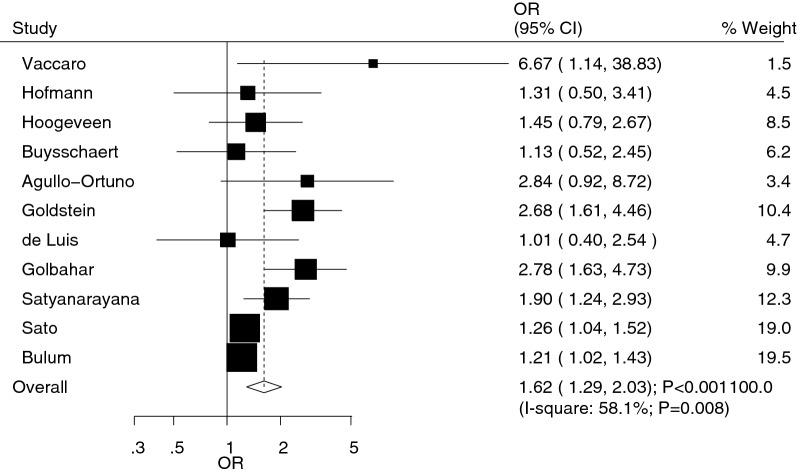
Association between homocysteine level and the risk of diabetic retinopathy

All included studies reported an association between Hcy and diabetic retinopathy. Pooled analysis results indicated that elevated Hcy level was associated with an increased risk of diabetic retinopathy (OR 1.62; 95% CI 1.29–2.03; p < 0.001; Fig. 2). Although substantial heterogeneity was observed in the magnitude of the effect across the studies (I^2^ = 58.1%; p = 0.008), the conclusion was not affected after sequential exclusion of any specific study from the pooled analyses (Table 2).

**Table 2:** Sensitivity analysis for diabetic retinopathy risk

Excluding study	OR and 95% CI	p value	Heterogeneity (%)	p value for heterogeneity
Vaccaro	1.57 (1.26–1.96)	< 0.001	56.7	0.014
Hofmann	1.64 (1.29–2.09)	< 0.001	62.3	0.005
Hoogeveen	1.64 (1.28–2.11)	< 0.001	62.3	0.005
Buysschaert	1.66 (1.31–2.12)	< 0.001	61.9	0.005
Agullo-Ortuno	1.58 (1.26–1.99)	< 0.001	59.6	0.008
Goldstein	1.50 (1.21–1.85)	< 0.001	47.2	0.048
de Luis	1.66 (1.31–2.11)	< 0.001	61.6	0.005
Golbahar	1.49 (1.21–1.84)	< 0.001	46.9	0.049
Satyanarayana	1.58 (1.24–2.02)	< 0.001	58.4	0.010
Sato	1.74 (1.30–2.34)	< 0.001	60.0	0.007
Bulum	1.75 (1.32–2.32)	< 0.001	54.5	0.019

*OR* odds ratio, *CI* confidence interval

Heterogeneity testing showed a value of p < 0.10 for diabetic retinopathy. Therefore, a meta-regression analysis was conducted based on sample size, mean age, percentage male, and BMI. Overall, sample size (p = 0.412), mean age (p = 0.892), percentage male (p = 0.659), and BMI (p = 0.452) did not significantly affect the relationship between Hcy and diabetic retinopathy (Additional file 1). The results of subgroup analysis indicated that this relationship in most subsets was consistent with the overall analysis (Table 3). However, elevated Hcy level was not associated with the risk of diabetic retinopathy if the sample size was < 100, the study included patients with type 1 diabetes mellitus, and the study was low quality.

**Table 3:** Subgroup analysis for diabetic retinopathy risk

Group	OR and 95% CI	*p* value	Heterogeneity (%)	*p* value for heterogeneity	Ratio between subgroups
Publication year					
Before 2005	1.89 (1.26–2.81)	0.002	31.0	0.203	1.29 (0.80–2.07)/0.300
2005 or after	1.47 (1.14–1.90)	0.003	66.2	0.019	
Country					
Eastern	2.26 (1.24–4.13)	0.008	81.4	0.001	1.73 (0.93–3.21)/0.085
Western	1.31 (1.12–1.52)	0.001	1.3	0.415	
Sample size					
≥ 100	1.66 (1.21–2.29)	0.002	66.9	0.006	0.99 (0.52–1.88)/0.971
< 100	1.68 (0.96–2.92)	0.067	42.9	0.154	
Mean age (years)					
≥ 60.0	1.47 (1.05–2.05)	0.025	50.6	0.088	0.77 (0.45–1.32)/0.350
< 60.0	1.90 (1.25–2.90)	0.003	68.2	0.008	
Percentage male (%)					
≥ 55.0	1.51 (1.04–2.21)	0.032	52.3	0.099	0.87 (0.52–1.48)/0.612
< 55.0	1.73 (1.20–2.50)	0.003	65.7	0.008	
BMI (kg/m^2^)					
≥ 25.0	1.35 (1.10–1.67)	0.004	45.1	0.105	0.75 (0.48–1.17)/0.211
< 25.0	1.79 (1.21–2.64)	0.004	0.0	0.488	
DM types					
1	1.27 (0.93–1.75)	0.134	17.3	0.305	0.79 (0.50–1.24)/0.306^a^
2	1.61 (1.16–2.23)	0.005	59.7	0.042	
Both	2.71 (1.70–4.30)	< 0.001	0.0	0.917	
Study quality					
High	1.70 (1.24–2.33)	0.001	66.0	0.007	1.06 (0.56–2.03)/0.854
Low	1.60 (0.91–2.82)	0.106	45.9	0.136	

*OR* odds ratio, *CI* confidence interval, *BMI* body mass index, *DM* diabetes mellitus

^a^Compared between type 1 and 2 DM

**Fig. 3:**
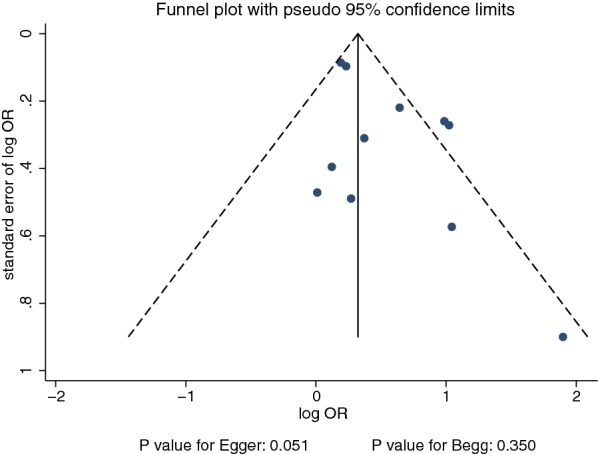
Funnel plot for diabetic retinopathy

The funnel plots did not rule out publication bias for diabetic retinopathy (Fig. 3). Although the Begg test showed no evidence of publication bias for diabetic retinopathy (p = 0.350), the Egger test showed potential evidence of publication bias for diabetic retinopathy (p = 0.051). The conclusions were not changed after adjustment for publication bias using the trim and fill method (OR 1.55; 95% CI 1.24–1.94; p < 0.001) [33].

## Discussion

Although many studies have indicated an association between Hcy and diabetic retinopathy, the results are conflicting [34, 35]. Among the many determinants of Hcy, the B-vitamin status was shown to be a major regulating factor; however, its relationship with diabetic retinopathy remains unclear [3]. This meta-analysis summarized 11 studies involving a total of 2184 diabetic patients from nine countries and regions in three continents. Elevated Hcy level was associated with an increased risk of diabetic retinopathy in diabetic patients. We are confident in this result, which was persistent after excluding any specific study. However, subgroup analysis indicated no significant difference between Hcy and diabetic retinopathy in studies with sample size < 100, participants with type 1 diabetes mellitus, and low quality.

Diabetic retinopathy is one of the most severe microvascular complications, which occurs due to metabolic disorders and endocrine system damage in diabetic patients [36]. The complex pathogenesis includes nonproliferative retinopathy (capillary swelling and deformation, blood-retinal barrier damage and retinal leakage, macular edema, and vision damage), followed by proliferative retinopathy (neovascularization, vitreous hemorrhage, retinal detachment, and eventually blindness) [37, 38]. Recently, the effects of oxidative stress and inflammation on the role of diabetic retinopathy have been extensively studied. Hcy is involved in oxidation–reduction reaction and induces oxidative stress. Plasma Hcy level is regulated by various factors, including genetic factors, nutrition, hormone levels, blood pressure, impaired renal function, and duration of diabetes [39, 40]. Hcy is implicated in vascular diseases via endotoxic and pro-proliferative effects, which could interfere with glutathione synthesis and methylation reactions [41]. Hcy promotes the occurrence and development of vascular diseases. Retinopathy was found to be prevalent in type 2 diabetes mellitus patients with higher Hcy concentrations. It may be a predictor of retinopathy in type 2 diabetes mellitus. However, whether plasma Hcy is involved in diabetic microvascular complications is not established [27]. The association between Hcy and type 1 diabetes mellitus has gained increasing attention. In the early stages of type 1 diabetes mellitus, patients with proper renal function and hyperfiltration may have lower plasma Hcy levels. The present study showed that elevated Hcy level significantly increased the progression of diabetic retinopathy, especially in type 2 diabetes mellitus. However, this relationship was not statistically significant in type 1 diabetes mellitus.

Elevated Hcy level and low folic acid level was observed in diabetic patients, which was associated with high risk of proliferative retinopathy [42]. The severity of diabetic retinopathy was found to be associated with lower folic acid and red blood cell folate levels [43]. Long-term use of metformin increased the risk of vitamin B12 and folate deficiency, and thus influenced Hcy metabolism and contributed to the progression of diabetic retinopathy [44]. A previous meta-analysis demonstrated hyperhomocysteinemia as a risk factor for the progression of diabetic retinopathy, especially for proliferative diabetic retinopathy. However, the results were based on continuous data, and a direct relationship was not confirmed [8]. Further, factor-difference for the relationship between Hcy level and diabetic retinopathy was not calculated. Therefore, the current updated meta-analysis was conducted to explore the association between Hcy and the risk of diabetic retinopathy.

The present study had several limitations. First, this analysis did not include studies in languages other than English or when the corresponding author could not be contacted. Second, the summary risk for diabetic retinopathy was highly heterogeneous, and the source of heterogeneity could not be detected by a subgroup analysis. The heterogeneity may be due to the differences in geographical regions (nine countries and regions in 11 studies), study designs and methods, and methodological qualities. Third, the adjusted models were different across the included studies, and these factors might play an important role in the development of diabetic retinopathy. Finally, subgroup analysis based on patients with no proliferative or proliferative diabetic retinopathy was not conducted due to these data were available in few studies.

## Conclusions

The present study demonstrated a strong positive correlation between plasma Hcy level and the progression of diabetic retinopathy. These data may help diabetic patients to achieve effective management in order to prevent the development and progression of diabetic retinopathy. Given the limitations of this study, further studies should be performed to confirm the findings and verify the effect of folic acid supplementation on the progression of diabetic retinopathy in diabetic patients.

## Additional file

**Additional file 1.** Meta-regression.

## Abbreviations

BMI: body mass index
CIs: confidence intervals
DM: diabetes mellitus
Hcy: homocysteine
NOS: Newcastle–Ottawa Quality Assessment Scale
OR: odds ratio

## References

[1] Resnikoff S, Pascolini D, Etya’ale D, Kocur I, Pararajasegaram R, Pokharel GP (2004). Global data on visual impairment in the year 2002. Bull World Health Organ.

[2] Kempen JH, O’Colmain BJ, Leske MC, Haffner SM, Klein R, Moss SE (2004). The prevalence of diabetic retinopathy among adults in the United States. Arch Ophthalmol.

[3] Satyanarayana A, Balakrishna N, Pitla S, Reddy PY, Mudili S, Lopamudra P (2011). Status of B-vitamins and homocysteine in diabetic retinopathy: association with vitamin-B12 deficiency and hyperhomocysteinemia. PLoS ONE.

[4] Zhang X, Saaddine JB, Chou CF, Cotch MF, Cheng YJ, Geiss LS (2010). Prevalence of diabetic retinopathy in the United States, 2005–2008. JAMA.

[5] Aydin E, Demir HD, Ozyurt H, Etikan I (2008). Association of plasma homocysteine and macular edema in type 2 diabetes mellitus. Eur J Ophthalmol.

[6] Elias AN, Eng S (2005). Homocysteine concentrations in patients with diabetes mellitus-relationship to microvascular and macrovascular disease. Diab Obes Metab.

[7] Chico A, Perez A, Cordoba A, Arcelus R, Carreras G, de Leiva A (1998). Plasma homocysteine is related to albumin excretion rate in patients with diabetes mellitus: a new link between diabetic nephropathy and cardiovascular disease?. Diabetologia.

[8] Xu C, Wu Y, Liu G, Liu X, Wang F, Yu J (2014). Relationship between homocysteine level and diabetic retinopathy: a systematic review and meta-analysis. Diagn Pathol..

[9] Coral K, Angayarkanni N, Gomathy N, Bharathselvi M, Pukhraj R, Rupak R (2009). Homocysteine levels in the vitreous of proliferative diabetic retinopathy and rhegmatogenous retinal detachment: its modulating role on lysyl oxidase. Invest Ophthalmol Vis Sci.

[10] Sun J, Xu Y, Zhu Y, Lu H, Deng H, Fan Y (2003). The relationship of methylenetetrahydrofolate reductase gene polymorphism and plasma homocysteine levels in type 2 diabetes mellitus patients with diabetic retinopathy. Zhonghua Yi Xue Yi Chuan Xue Za Zhi.

[11] Agardh E, Hultberg B, Agardh CD (2000). Severe retinopathy in type 1 diabetic patients is not related to the level of plasma homocysteine. Scand J Clin Lab Invest.

[12] Hultberg B, Agardh E, Andersson A, Brattstrom L, Isaksson A, Israelsson B (1991). Increased levels of plasma homocysteine are associated with nephropathy, but not severe retinopathy in type 1 diabetes mellitus. Scand J Clin Lab Invest.

[13] Moher D, Liberati A, Tetzlaff J, Altman DG (2009). Preferred reporting items for systematic reviews and meta-analyses: the PRISMA statement. PLoS Med..

[14] Stang A (2010). Critical evaluation of the Newcastle–Ottawa scale for the assessment of the quality of nonrandomized studies in meta-analyses. Eur J Epidemiol.

[15] DerSimonian R, Laird N (1986). Meta-analysis in clinical trials. Control Clin Trials.

[16] Ades AE, Lu G, Higgins JP (2005). The interpretation of random-effects meta-analysis in decision models. Med Decis Making.

[17] Deeks JJ, Higgins J, Altman DG (2008). Analysing data and undertaking meta-analyses. Cochrane handbook for systematic reviews of interventions: Cochrane book series.

[18] Tobias A (1999). Assessing the influence of a single study in meta-analysis. Stata Tech Bull.

[19] Thompson SG, Higgins JP (2002). How should meta-regression analyses be undertaken and interpreted?. Stat Med.

[20] Altman DG, Bland JM (2003). Interaction revisited: the difference between two estimates. BMJ.

[21] Egger M, Davey Smith G, Schneider M, Minder C (1997). Bias in meta-analysis detected by a simple, graphical test. BMJ.

[22] Begg CB, Mazumdar M (1994). Operating characteristics of a rank correlation test for publication bias. Biometrics.

[23] Vaccaro O, Ingrosso D, Rivellese A, Greco G, Riccardi G (1997). Moderate hyperhomocysteinaemia and retinopathy in insulin-dependent diabetes. Lancet.

[24] Hofmann MA, Kohl B, Zumbach MS, Borcea V, Bierhaus A, Henkels M (1998). Hyperhomocyst(e)inemia and endothelial dysfunction in IDDM. Diab Care.

[25] Hoogeveen EK, Kostense PJ, Eysink PE, Polak BC, Beks PJ, Jakobs C (2000). Hyperhomocysteinemia is associated with the presence of retinopathy in type 2 diabetes mellitus: the Hoorn study. Arch Intern Med.

[26] Buysschaert M, Dramais AS, Wallemacq PE, Hermans MP (2000). Hyperhomocysteinemia in type 2 diabetes: relationship to macroangiopathy, nephropathy, and insulin resistance. Diab Care.

[27] Agullo-Ortuno MT, Albaladejo MD, Parra S, Rodriguez-Manotas M, Fenollar M, Ruiz-Espejo F (2002). Plasmatic homocysteine concentration and its relationship with complications associated to diabetes mellitus. Clin Chim Acta.

[28] Goldstein M, Leibovitch I, Yeffimov I, Gavendo S, Sela BA, Loewenstein A (2004). Hyperhomocysteinemia in patients with diabetes mellitus with and without diabetic retinopathy. Eye (Lond)..

[29] de Luis DA, Fernandez N, Arranz ML, Aller R, Izaola O, Romero E (2005). Total homocysteine levels relation with chronic complications of diabetes, body composition, and other cardiovascular risk factors in a population of patients with diabetes mellitus type 2. J Diab Complications.

[30] Golbahar J, Rahimi M, Tabei MB, Aminzadeh MA (2008). Clinical risk factors and association of hyperhomocysteinemia with diabetic retinopathy in Iranian type 2 diabetes patients: a cross-sectional study from Shiraz, Southern Iran. Diab Metab Syndr.

[31] Sato Y, Ouchi K, Funase Y, Yamauchi K, Aizawa T (2013). Relationship between metformin use, vitamin B12 deficiency, hyperhomocysteinemia and vascular complications in patients with type 2 diabetes. Endocr J.

[32] Bulum T, Blaslov K, Duvnjak L (2016). Plasma homocysteine is associated with retinopathy in type 1 diabetic patients in the absence of nephropathy. Semin Ophthalmol..

[33] Duval S, Tweedie R (2000). A nonparametric “trim and fill” method of accounting for publication bias in meta-analysis. J Am Stat Assoc.

[34] Abdella NA, Mojiminiyi OA, Akanji AO, Moussa MA (2002). Associations of plasma homocysteine concentration in subjects with type 2 diabetes mellitus. Acta Diabetol.

[35] Agardh CD, Agardh E, Andersson A, Hultberg B (1994). Lack of association between plasma homocysteine levels and microangiopathy in type 1 diabetes mellitus. Scand J Clin Lab Invest.

[36] Stanford MR (2004). The pathogenesis of diabetic retinopathy. Br J Ophthalmol.

[37] Pan HZ, Zhang H, Chang D, Li H, Sui H (2008). The change of oxidative stress products in diabetes mellitus and diabetic retinopathy. Br J Ophthalmol.

[38] Trunov A, Chernykh D, Chernykh V (2014). Intraocular cytokines and growth factors imbalance in pathogenesis of proliferative diabetic retinopathy. Ophthalmologica Journal international d’ophtalmologie Int J Ophthalmol Zeitschrift fur Augenheilkunde.

[39] Saeed BO, Nixon SJ, White AJ, Summerfield GP, Skillen AW, Weaver JU (2004). Fasting homocysteine levels in adults with type 1 diabetes and retinopathy. Clin Chim Acta.

[40] Lim CP, Loo AV, Khaw KW, Sthaneshwar P, Khang TF, Hassan M (2012). Plasma, aqueous and vitreous homocysteine levels in proliferative diabetic retinopathy. Br J Ophthalmol.

[41] Chen C, Conklin BS, Ren Z, Zhong DS (2002). Homocysteine decreases endothelium-dependent vasorelaxation in porcine arteries. J Surg Res.

[42] Ayata A, Yildirim Y, Ozcan O (2012). Comment on: ‘Plasma, aqueous and vitreous homocysteine levels in proliferative diabetic retinopathy’. Br J Ophthalmol.

[43] Malaguarnera G, Gagliano C, Salomone S, Giordano M, Bucolo C, Pappalardo A (2015). Folate status in type 2 diabetic patients with and without retinopathy. Clin Ophthalmol..

[44] Nurpeisov V (2012). A58: long term treatment with metformin in patients with type 2 diabetes and risk of vitamin B-12 deficiency. J Am Geriatr Soc.

