# Supplementary material for: Association between homocysteine level and the risk of diabetic retinopathy: a systematic review and meta-analysis
Source: Diabetol Metab Syndr. 2018 Aug 2;10:61. doi: 10.1186/s13098-018-0362-1 (PMC6071377; doi:10.1186/s13098-018-0362-1)
Supplement: Supplementary file 1 — Additional file 1. Meta-regression. [file 13098_2018_362_MOESM1_ESM.docx]

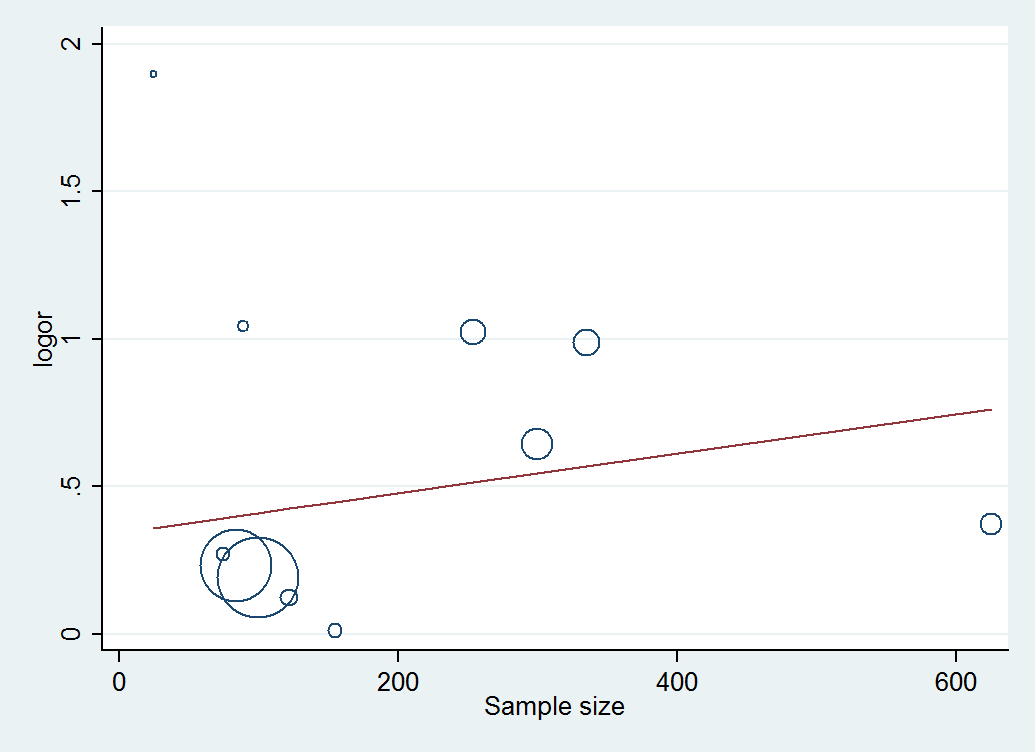


Figure S1. Meta-regression analysis based on sample size (p=0.412).


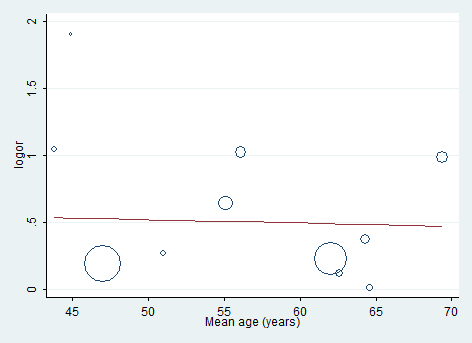


Figure S2. Meta-regression analysis based on mean age (p=0.892).


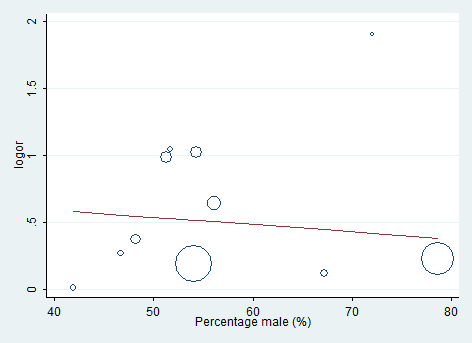


Figure S3. Meta-regression analysis based on percentage male (p=0.659).


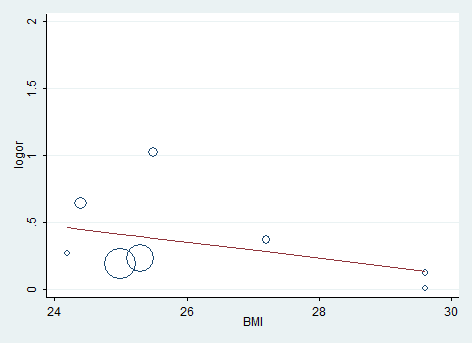


Figure S4. Meta-regression analysis based on BMI (p=0.452).
